# Use of a catch-up programme to improve routine immunization in 13 provinces of Papua New Guinea, 2020–2022

**DOI:** 10.5365/wpsar.2023.14.4.1055

**Published:** 2023-12-19

**Authors:** Dessie Ayalew Mekonnen, Mathias Bauri, Martha Pogo, Mei Shang, Deborah Bettels, Shaikh Humayun Kabir, Waramin Edward, Bieb Sibauk, Milena Dalton, Geoff Miller, Ananda Amarasinghe, Yoshihiro Takashima, Dapeng Luo, Sevil Huseynova

**Affiliations:** a World Health Organization Representative Office for Papua New Guinea, Port Moresby, Papua New Guinea.; b National Department of Health, Port Moresby, Papua New Guinea.; c World Health Organization, Geneva, Switzerland.; d World Health Organization Representative Office for Afghanistan, Kabul, Afghanistan.; e United Nations International Children’s Emergency Fund, New York City, New York, United States of America.; f Burnet Institute, Melbourne, Victoria, Australia.; g Papua New Guinea–Australia Transition to Health, Department of Foreign Affairs and Trade, Barton, Australian Capital Territory, Australia.; h World Health Organization Regional Office for the Western Pacific, Manila, Philippines.

## Abstract

**Objective:**

Routine immunization coverage in Papua New Guinea has decreased in the past 5 years. This persistently low routine immunization coverage has resulted in low population immunity and frequent outbreaks of vaccine-preventable disease across the country. We describe the use of a catch-up programme to improve routine immunization during the coronavirus disease pandemic in Papua New Guinea during 2020–2022.

**Methods:**

In June 2020, 13 provinces of Papua New Guinea were selected to undergo a vaccination catch-up programme, with technical support from the World Health Organization (WHO) and the United Nations Children’s Fund. Twelve provinces received financial and logistic support through the Accelerated Immunization and Health Systems Strengthening programme, and one received support from WHO. All stakeholders were involved in planning and implementing the catch-up programme.

**Results:**

Between July 2020 and June 2022, about 340 health facilities conducted catch-up activities. The highest number of children aged under 1 year were vaccinated in 2022 (*n* = 33 652 for third dose of pentavalent vaccine). The national coverage of routine immunization (including the catch-up vaccinations) increased between 2019 and 2020 – by 5% for the third dose of pentavalent vaccine, 11% for the measles-rubella vaccine and 16% for the inactivated poliovirus vaccine. The coverage declined slightly in 2021 before increasing again in 2022.

**Discussion:**

The catch-up programme was an instrumental tool to improve routine immunization coverage between 2020 and 2022 and during the pandemic in Papua New Guinea. With appropriate technical and logistic support, including financial and human resources, catch-up programmes can strengthen routine immunization coverage across the country.

Immunization is a cost-effective public health programme and a key contributor to improving health. (*1*) Globally, in 2021, more than 25 million infants were reported to have never been vaccinated or to have been underimmunized. Most of these children tend to live in communities in low- and middle-income countries that have never received routine immunization services, including Papua New Guinea. These communities lack access to vaccination services, resulting in an increased risk of outbreaks of vaccine-preventable disease. This risk has been exacerbated by disruptions associated with the coronavirus disease (COVID-19) pandemic. (*2*)

The Papua New Guinea National Immunization Strategy 2021–2025 described a “catastrophic” situation with immunization; for example, national coverage of the third dose of pentavalent vaccine decreased from 70% in the 2000s to less than 45% during 2016–2020. (*3*) This persistently low routine immunization coverage led to low population immunity, and was the underlying cause of several outbreaks of vaccine-preventable disease across the country. For example, a measles outbreak was reported in 2013–2014; it affected all 22 provinces and resulted in 2299 laboratory-confirmed cases, and continued with local and small-scale measles outbreaks reported in several provinces. (*4*) A study conducted in East Sepik province of Papua New Guinea in 2020 found that the prevalence rates of anti-measles and rubella IgG were 63% and 82%, respectively. (*5*) An outbreak of circulating vaccine-derived type 1 poliovirus occurred in 2018, with 26 cases confirmed in nine of the 22 provinces. (*6*) In March 2020, the country confirmed the first imported COVID-19 case; by 21 February 2023, there were 46 792 confirmed cases including 670 deaths reported. (*7*)

In a cross-sectional study conducted in East New Britain province, contributing factors for low immunization coverage included a lack of local planning based on locations of child populations, limited intensification of outreach services, incomplete local information and lack of trained human resources. (*8*) Another study found that there were several barriers to vaccine delivery, including lack of access to health-care services, natural disasters and intertribal conflicts. (*9*) In 2020, immunization service delivery was negatively affected by the COVID-19 pandemic because the government issued strict movement restrictions that resulted in reduced health clinic attendance and outreach visits by health-care workers. In 2021, the introduction of the COVID-19 vaccine also negatively affected routine immunization services because the limited health-care workforce and fragile health system were overwhelmed with COVID-19 vaccination activities. This report describes the use of a catch-up programme to improve routine immunization during the COVID-19 pandemic in Papua New Guinea in 2020–2022.

## Methods

### Study area

**Map 1:**
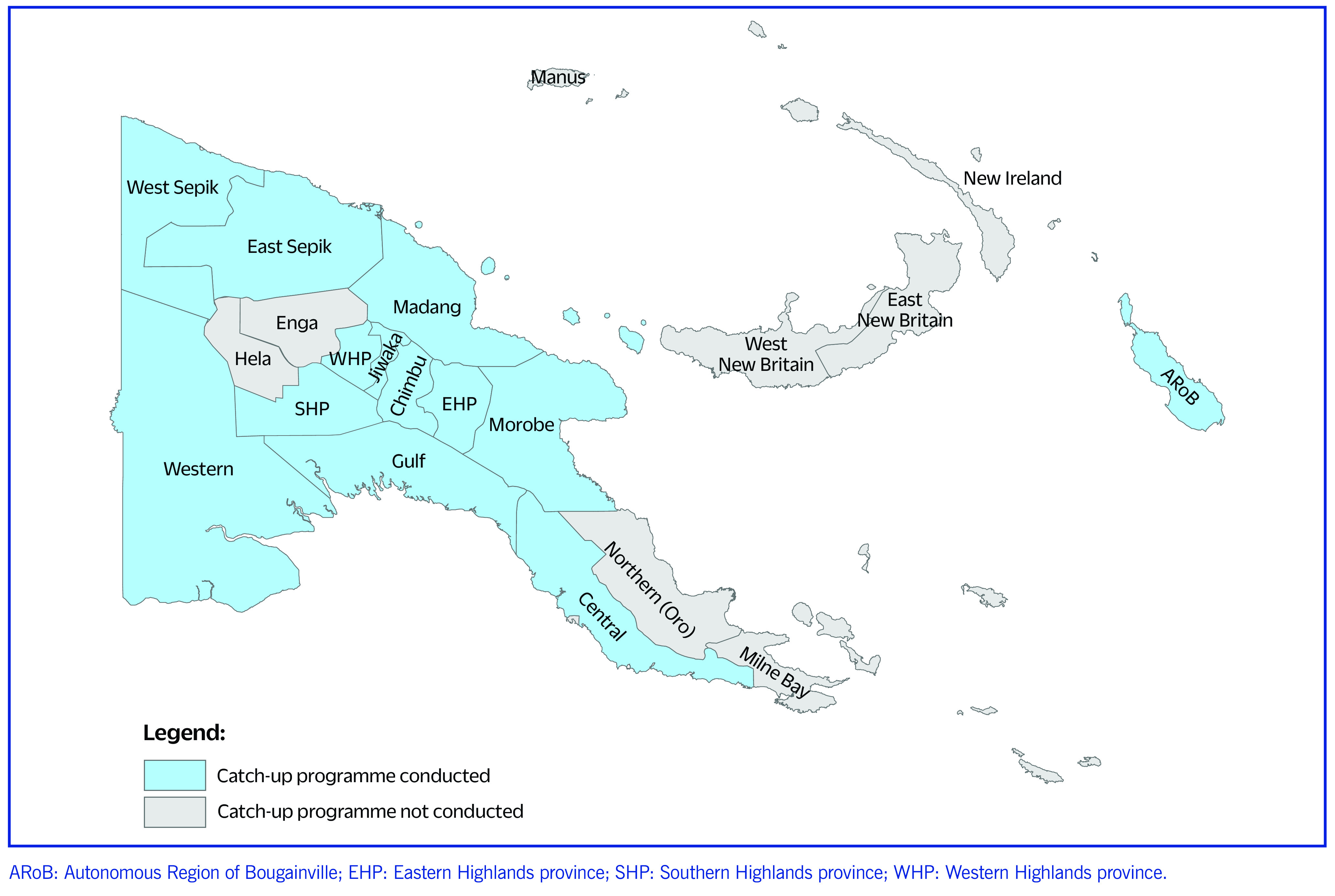
The 13 provinces of Papua New Guinea in which the immunization catch-up programme was conducted, 2020–2022

Papua New Guinea has 22 provinces, 89 districts and 349 local-level governments, with over 750 health facilities delivering routine immunization services across the country and 533 health facilities in 13 provinces. According to the 2011 census, the projected population for 2022 was 9 593 926. The target number of children for vaccination aged under 1 year is 314 667, with 70% of these children residing in 13 provinces (**Map 1**).

[**insert** Map 1]

### Design of the catch-up programme

The catch-up programme was designed to provide an additional three rounds of mobile and outreach vaccination activities over 2 weeks of each year in each province. Outreach involved the vaccination team staying overnight for 3–5 days in remote villages; in contrast, mobile teams returned on the same day. Vaccinations were provided to children who had received no doses and underimmunized children aged under 2 years. All available vaccines, in accordance with the national immunization schedule, (*10*) were offered during the catch-up programme except for the hepatitis B birth dose.

The catch-up programme was led by the Papua New Guinea National Department of Health (NDOH), with technical support from the World Health Organization (WHO) and the United Nations Children’s Fund (UNICEF). Financial and logistic support was provided to 12 provinces through the Accelerated Immunization and Health Systems Strengthening Programme, which was donated by GAVI, the Vaccine Alliance, and by the governments of Australia and New Zealand; WHO provided technical support to all 13 provinces and financial support to one province. All stakeholders were involved in planning and implementing the catch-up programme. The NDOH conducted the immunization catch-up programme in 13 provinces from July 2020 to June 2022. In July 2020, the first virtual meeting was conducted, attended by all stakeholders, and the objectives and planning of the catch-up programme were discussed. Meetings continued monthly to review the performance of the catch-up programme.

### Data collection and analysis

The country’s existing electronic national health information system (eNHIS) was used to collect immunization data. The data were submitted to the WHO/UNICEF Estimates of National Immunization Coverage (WUENIC) database annually. Unlike the eNHIS data, the WUENIC data are publicly available online and thus could be referenced for the coverage in this manuscript. A simple Microsoft Excel database was created to monitor the catch-up programme vaccinations in the field. The data were analysed for children aged under 1 year only (even though the catch-up programme was for children aged under 2 years), because complete data were only available for those aged under 1 year. Vaccines included in the analysis were bacille Calmette–Guérin (BCG), first and third doses of pentavalent vaccine (Penta1 and Penta3), first dose of measles-rubella vaccine (MR1), oral poliovirus vaccine (OPV) and inactivated poliovirus vaccine (IPV). The three doses of pentavalent vaccine offer protection against diphtheria, tetanus, pertussis, hepatitis B and *Haemophilus influenza* type B.

## Results

About 340 health facilities in the 13 provinces conducted at least three rounds of catch-up programme activities in 2020, two rounds in 2021 and four rounds in 2022.

The highest number of children aged under 1 year vaccinated during the catch-up programme was in 2022, with 40 897 vaccinated with Penta1, 33 652 with Penta3 and 37 099 with MR1 (**Table 1**). This is likely because there were four rounds of catch-up programme activities in 2022, and these started in February.

**Table 1:** Total target children and number of children aged under 1 year vaccinated during the catch-up programme activities in 13 provinces of Papua New Guinea, 2020–2022

Province	2020				2021				2022			
	Target < 1 year	Penta1	Penta2	MR1	Target < 1 year	Penta1	Penta3	MR1	Target < 1 year	Penta1	Penta3	MR1
ARoB	10 830	1480	1186	1687	11 164	1156	288	401	11 508	1089	694	639
Madang	26 606	5747	2842	4499	27 471	385	865	1275	28 364	1507	679	1416
East Sepik	23 263	3970	3150	3523	23 959	439	2359	2287	24 675	1720	1499	1514
West Sepik	11 224	2442	2037	2450	11 505	686	1129	1589	11 792	6808	6285	7451
Simbu	10 432	170	181	358	10 609	257	784	996	10 790	4077	3746	3786
Jiwaka	11 334	728	661	674	11 666	2723	864	870	12 008	3296	3711	2556
Southern Highlands	21 691	2839	2920	4043	22 299	1123	891	1341	22 923	4249	3911	5003
Western Highlands	13 806	1173	1070	406	14 166	2125	785	563	14 536	2303	1783	1268
Eastern Highlands	21 654	2475	2634	2911	22 165	856	729	773	22 688	5961	4170	4905
Central	10 177	0	0	0	10 439	261	675	724	10 707	3880	3201	3838
Gulf	7211	195	164	165	7407	0	0	0	7608	686	387	476
Western	11 303	0	0	0	11 665	853	82	318	12 038	864	736	752
Morobe	29 377	1870	1278	2512	30 123	1230	311	390	30 888	4457	2850	3495
**Total**	**208 909**	**23 089**	**18 123**	**23 228**	**214 637**	**12 094**	**9762**	**11 527**	**220 526**	**40 897**	**33 652**	**37 099**

ARoB: Autonomous Region of Bougainville; MR1: first dose of measles-rubella vaccine; Penta1: first dose of pentavalent vaccine; Penta2: second dose of pentavalent vaccine; Penta3: third dose of pentavalent vaccine.

The performance of catch-up programme activities varied across the provinces. In 2020–2021, East Sepik province vaccinated the highest number of children for Penta3 and MR1, whereas in 2020, Madang province vaccinated the highest number of children for MR1. West Sepik demonstrated the highest number of children vaccinated for Penta3 and MR1 in 2022. Gulf province vaccinated the lowest number of children through catch-up programme activities during the 2 years (**Table 1**).

MR1 and first dose of IPV (IPV1) accounted for the highest number of doses administered during the catch-up programme compared with other vaccines. BCG had the lowest number of doses administered during the catch-up programme (**Supplementary Fig. 1**).

**Fig. 1:**
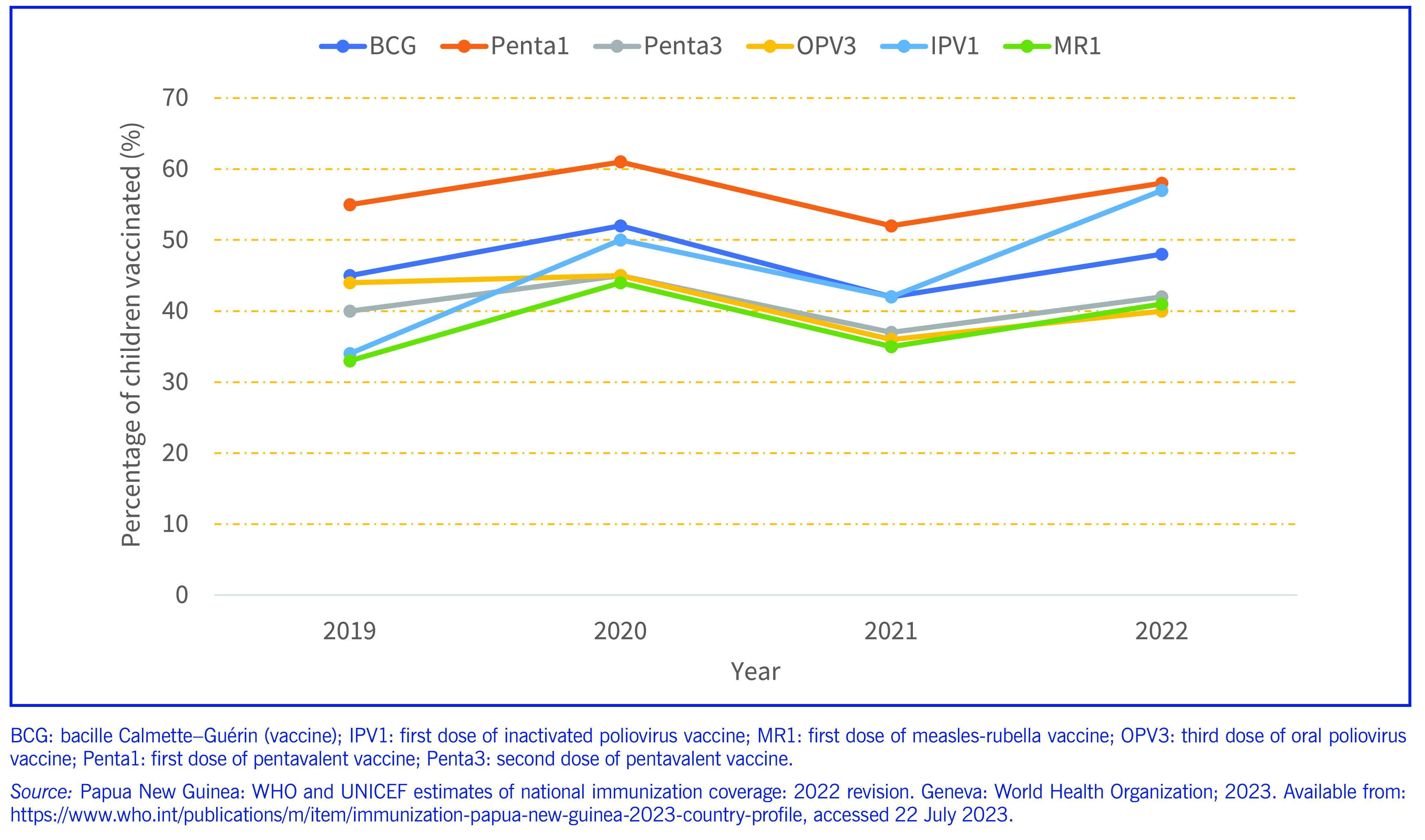
National routine immunization coverage of children aged under 1 year, Papua New Guinea, 2019–2022

[**insert** Figure 1]

The national immunization coverage of Penta3, MR1 and IPV1 increased from 40%, 33% and 34% in 2019 to 45%, 44% and 50% in 2020, respectively. The increases ranged from 5% (14 927 additional children aged

under 1 year completed Penta3) to 11% (32 839 children aged under 1 year received MR1) and 16% (47 765 children aged under 1 year received IPV1). This improvement was probably due to the catch-up programme activities in the 13 provinces. In 2021, the coverage was slightly lower than in 2019, 2020 and 2022 for some vaccines (**Fig. 1**). The coverage of Penta3, MR1 and IPV1 increased from 37%, 35% and 42% in 2021 to 42%, 41% and 57% in 2022, respectively (**Fig. 1**). The dropout rate from Penta1 to Penta3 was over 10%.

## Discussion

National routine immunization coverage improved in Papua New Guinea between 2019 and 2020, slightly declined in 2021 and increased again in 2022. These changes were probably due to the implementation of the catch-up programme in 13 provinces. The decline in coverage in 2021 was likely due to the introduction of COVID-19 vaccination at a point when only two rounds of catch-up programme activities had been conducted. The COVID-19 pandemic had a negative effect on routine immunization in many countries, especially in the initial pandemic phases. This highlights the importance of maintaining and recovering routine immunization through periodic catch-up programmes during and after a pandemic. (*11*)

Several key field observations were made during the implementation of the catch-up programme. Lessons identified about what is essential for the success of catch-up programme activities included making adequate logistic and financial preparations (e.g. through effective coordination among partners) before starting implementation of activities, including obtaining the average estimated cost per round per province of US$ 10 000; active engagement from district health managers and officers in charge during the planning and implementation stages of the catch-up programme activities; technical support and close monitoring from WHO subnational consultants, to ensure the implementation of a quality microplan; human resources, financial support and timely disbursement of funds at the health facility level (critical for improving the immunization coverage); and distribution and availability of vaccines at the health facility level. A similar study revealed that logistic availability, adequate staffing and reallocation of resources during the COVID-19 pandemic are key elements for the success of a routine immunization catch-up programme. (*12*)

There were several limitations to conducting the catch-up vaccination programme, including a shortage of health-care workers and a lack of resources such as laptops and computers, electricity supply and Internet connection, particularly in remote areas. This limited the data collection to those aged under 1 year because the full data set was not available for those aged under 2 years.

During the pandemic, the dropout rate in Papua New Guinea from Penta1 to Penta3 was higher than the recommended 10%. Intensive efforts need to be made to ensure effective communication during the first immunization visit, as this is vital for ensuring timely administration of the second dose and completion of the recommended dose schedule.

The catch-up programme was instrumental in improving routine immunization coverage within a short period of time in Papua New Guinea. It is recommended that similar catch-up programmes be part of the country’s national immunization programme, with four rounds implemented each year and funding of US$ 10 000 allocated per round per province. With appropriate technical and logistic support, including finances and human resources, the catch-up programme can contribute to the effort to strengthen routine immunization coverage across Papua New Guinea.
